# A High-Throughput Screening Strategy for *Bacillus subtilis* Producing Menaquinone-7 Based on Fluorescence-Activated Cell Sorting

**DOI:** 10.3390/microorganisms13030536

**Published:** 2025-02-27

**Authors:** Lina Yang, Can Tang, Yan Cui, Jianhua Zhang

**Affiliations:** 1 School of Agriculture and Biology, Bor S. Luh Food Safety Research Center, Shanghai Jiao Tong University, Shanghai 200240, China; yang_ln@sjtu.edu.cn (L.Y.); tangcan01@sjtu.edu.cn (C.T.); cyan9028@163.com (Y.C.); 2 NMPA Key Laboratory for Testing Technology of Pharmaceutical, Shanghai Institute for Food and Drug Control, Shanghai 201203, China

**Keywords:** vitamin K_2_, ARTP mutant libraries, high yield mutant selection, fluorescence intensity, resequencing

## Abstract

Menaquinone-7 (MK-7) is recognized for its important biological activity, and *Bacillus subtilis* is the preferred strain for its fermentative production. However, the limited phenotypic diversity among high-yielding strains complicates the development of rapid screening methods. To address this, we utilized the effect of MK-7 on transmembrane potential to develop a high-throughput screening (HTS) strategy for efficiently identifying strains with improved MK-7 production. Among various membrane potential fluorescent dyes tested, Rhodamine 123 was selected for quantifying intracellular MK-7 levels due to its effective staining and minimal impact on cell growth. By optimizing pretreatment protocols and staining conditions, we established an HTS protocol that combines fluorescence-activated cell sorting with HPLC to identify strains with increased MK-7 production. A linear correlation was observed between mean MK-7 content and average fluorescence intensity (R^2^ = 0.9646). This approach was applied to mutant libraries generated through atmospheric room temperature plasma mutagenesis. After three cycles of mutagenesis and screening, the mutant AR03-27 was identified, showing an 85.65% increase in MK-7 yield compared to the original SJTU2 strain. Resequencing analysis revealed that the top three mutants contained mutations in genes related to membrane transport, suggesting their potential role in enhancing MK-7 yield.

## 1. Introduction

Vitamin K_2_, also known as menaquinone (MK), is one of three forms of vitamin K, characterized by a naphthoquinone ring and an isoprene side chain. MK varieties are categorized based on the number of isoprene units in the structure, resulting in 10 types labeled as MK-n (where n ranges from 4 to 13) [1]. Among these, menaquinone-7 (MK-7) notable for its superior biological activity [2] and plays a crucial role in preventing or alleviating cardiovascular diseases, osteoporosis, diabetes, cancer, and Alzheimer’s disease [3]. This highlights its significant therapeutic potential and promising market prospects.

Humans can only acquire MK externally, which is commonly produced by three methods: extraction from food, chemical synthesis, and microbial fermentation. Foods such as natto [4], honey [5], and yogurt [6] contain trace amounts of MK-7. However, due to its low content and the high cost of extraction, deriving MK-7 from food sources is not economically viable for industrial purposes.

Chemical synthesis of MK-7 predominantly yields cis-isomers [7], which have low biological activity and involve a complex synthesis process. This method also generates numerous by-products and contributes to environmental pollution. In contrast, producing MK-7 through microbial fermentation addresses these issues of low activity and severe reaction conditions. Consequently, this method has emerged as a major focus of current research. Many microorganisms are capable of producing MK, a crucial electron carrier in the respiratory chain [8]. *Bacillus subtilis* is extensively utilized for the industrial production of MK because it is generally recognized as safe (GRAS) [9], exhibits a high growth rate [10]) and is relatively easy to genetically manipulate [11]. Notably, MK-7 produced by *B. subtilis* can account for over 90% of the total MK production [12], establishing it as the preferred species for MK-7 fermentation.

Since no phenotypic diversity has been observed in high-yielding MK-7 strains [13], it is challenging to rapidly screen large mutant libraries generated by genetic engineering or traditional breeding methods. Currently, screening is still conducted individually on a large scale using the anti-structural analogs plate method [14], which is characterized by low throughput, slow processing speed, and huge workload, all contributing to inefficient screening [15].

As a key component of the electron transport chain, MK significantly impacts the membrane potential [16], the voltage difference across the cell membrane, which is a critical physiological characteristic influencing various biological processes [17]. Research has demonstrated that membrane potential drops from −48 mV to −200 mV, when mitochondria are exposed to ultraviolet radiation, leading to the inactivation of MK-7 [18]. Fluorescent dyes for membrane potential are small molecule compounds whose intensity changes in response to variations in membrane voltage. These dyes are commonly used to assess the intima potential and detect changes in membrane potential. Consequently, their fluorescence intensity (FI) can serve as an indicator of MK-7 levels, potentially aiding in the quantification of MK-7 in bacteria. The fluorescent dyes, such as bis-(1, 3-dibutylbarbituric acid) trimethylene oxonol (DiBAC4 (3)) [19], 3, 3′-dihexyloxacarbocyanine iodide (DiOC6 (3)) [20], Tetramethyl rhodamine ethyl ester and Rhodamine 123 (Rh123) [21], reflect the change in membrane potential. The FI of Rh123 has been linked to MK content of *Flavobacterium* sp. [22], and DiOC6 (3) was found to be well bound to all germinated *B. subtilis* spores, but not to dormant spores without membrane potential [23]. Therefore, a staining protocol that can accurately measure MK content in a combinatorial screen must allow the dye to enter the cell and maintain high viability.

Despite these insights, a reliable high-throughput screening (HTS) method for identifying high-yielding MK-7 strains in *B. subtilis* mutant libraries is still lacking. Developing a robust HTS method for *B. subtilis* strains that produce MK-7 is crucial to address the challenges in strain selection and could enhance industrial MK production.

Over the past decade, microfluidic technology has emerged as a key tool in HTS, offering advantages such as rapid processing, low cost, high automation, and efficient screening of target products [24]. There are several types of active microfluidic devices including fluorescence-activated cell sorting (FACS), magnetic-activated cell sorting (MACS), dielectrophoresis (DEP), and the acoustic microfluidic system. Among these, FACS has become the most widely used and desirable cell sorting technique due to its high accuracy, efficiency, and precision [25]. The FACS system can simultaneously analyze multiple parameters, such as cell size, structure, and fluorescence signals, with the capability to sort and count over 100,000 cells per second. It is equipped with detectors capable of measuring 14 to 17 different fluorescent labels [26].

In this study, we developed an HTS method for identifying MK-7-producing *B. subtilis* strains and applied it for the first time to screen a large number of ARTP mutants. Integrated with HPLC, this method enabled the identification of high-yielding mutants with enhanced MK-7 production, overcoming the inefficiencies and labor-intensive nature of traditional plate-based screening methods. Furthermore, we conducted a genetic analysis of the original strain and the high-yielding MK-7 mutants through resequencing, revealing key genetic differences.

## 2. Materials and Methods

### 2.1. Strains and Cultural Conditions

The strain *B. subtilis* SJTU2 (CGMCC 2801) was isolated and preserved in our laboratory [27]. The strain was initially cultured at 37 °C in Luria-Bertani (LB) medium (Oxoid Ltd., Basingstoke, UK), consisting of 10 g/L tryptone, 5 g/L yeast extract, and 5 g/L NaCl. Following overnight cultivation, the strain was inoculated into a fermentation medium and cultivated at 37 °C for 72 h. To verify the correlation between MK-7 content and FI, five distinct culture protocols that significantly varied the MK-7 yield were designed, as detailed in Table 1.

### 2.2. Pretreatment of Cells Before Staining

After 72 h of cultivation, the cell cultures were harvested following the modified method of Lee [33]. The cells were resuspended in one of the following solutions: 1 mL 70% (*w*/*v*) isopropanol, 1 mL 1% (*w*/*v*) dimethyl sulfoxide (DMSO, Sinopharm chemical Reagent Co., Ltd., Shanghai, China), 1 mL ice-cold TSE buffer (10% (*w*/*v*) sucrose (Aladdin, Shanghai, China), 10 mM Tris-HCl (pH7.5) (Sigma-Aldrich, St. Louis, MO, USA), 0.5 mM EDTA (Sangon Biotech, Shanghai, China), or 1 mL ice-cold ETM buffer (0.5 M sorbitol, 0.5 M mannitol (Aladdin), 10% glycerol (Aladdin)). Each mixture was then incubated on ice for 10 min.

Heat shock was employed as a specialized pretreatment method. The process involved, resuspending the centrifuged cells in 1 mL of ice-cooled 0.1 mM CaCl_2_-MgCl_2_ (Sinopharm) solution and incubating them on ice for 10 min. The sample was then centrifuged (10 min, 5500× *g*, 4 °C) and resuspended in 1 mL of 0.1 M CaCl_2_ containing 5 μL of 1 mg/mL Rh123 (Sigma-Aldrich) solution. Following vigorous vortex mixing, the cells were subjected to a 90-s heat shock at 42 °C and then incubated in the dark for 10 min [34]. Finally, the cells were centrifuged and washed twice with ice-cold distilled deionized water (DDW) before analysis by FACS. The treatment that results in the greatest increase in FI is regarded as optimal.

### 2.3. Optimization of Staining Conditions for Pretreated Cells

The stock solutions for three dyes were prepared in DMSO as follows: DiBAC4 (3) (Aladdin, Shanghai, China) and DiOC6 (3) (Sigma-Aldrich) at 5 mM, Rh123 at 1 mg/mL.

Following cell pretreatment and a 10 min ice incubation, the cells were centrifuged (10 min, 5500× *g*, 4 °C) and resuspended in 1 mL of ice-cold solution (choices included DDW, PBS (Sangon Biotech), 0.9% NaCl (Sinopharm) or 1 mM MgCl_2_). The mixtures were vigorously vortexed with different concentrations of the dyes: 1 μmol/L of DiBAC4 (3), 5 μg/mL of Rh 123, and 40 nmol/L for DiOC6 (3). The mixtures were incubated in the dark at room temperature for different periods before another centrifugation, followed by two washes with ice-cold DDW. FACS was then performed to analyze the samples. After different staining times (10, 20, 30, 40, 50, and 60 min), the FI of the cells was measured by FACS to assess their stability.

### 2.4. FACS for Average FI

FACS was performed to measure the average FI of the cells with an Aria Ⅱ system (Becton-Dickinson, Mountain View, CA, USA). For cells stained with DiOC6 (3), DiBAC4 (3) and Rh 123, air-cooled argon ion lasers were excited at 488 [22], 490 and 507 nm, respectively. The emitted fluorescence was detected at 525 ± 40 nm using the FL-1 fluorescence channel for specific fluorescence monitoring. The sorting rate was set at 2000 cells per second. The average FI of the samples was subsequently analyzed digitally using FlowJo 10.8 software, which was then examined for its relationship with MK-7 content. The unstained cells were used as a control.

### 2.5. Quantification of MK-7 by HPLC

The fermentation solution was collected under different conditions, and the OD_600_ of each sample was adjusted to 1.0. Cells in ten milliliters were harvested by centrifugation. The cells were freeze-dried and MK-7 was extracted by adding 5 mL of a methanol: dichloromethane solution (9:1, *v*/*v*), and oscillating at 55–60 °C for 20 min. After centrifugation, the upper organic phase was collected and stored for analysis.

The quantification of MK-7 was performed using an HPLC system (Alliance e2695, Waters, Milford, MA, USA) equipped with a Shimadzu C18 column (250 × 4.6 mm, 5 μm) maintained at 40 °C. Methanol-dichloromethane (9:1, *v*/*v*) was used as the mobile phase at a flow rate of 1.0 mL/min. A wavelength of 254 nm was used for calibration and analysis. Pure MK-7 (Sigma-Aldrich) was used as a standard for calibration and quantification.

### 2.6. The Correlation Between MK-7 Contents and Fluorescence Intensity

To verify the correlation between MK-7 content and FI, the cells cultured with five distinct protocols were stained under the optimized conditions, and the MK-7 yield was assayed. The correlation between the mean MK-7 yields and the average FI values was calculated.

### 2.7. ARTP Mutagenesis

The parent strain culture and the ARTP mutagenesis were performed following the method of Li [35], with modifications. After an 80 s ARTP treatment (radiofrequency power: 120 W), all mutant bacterial fluids were inoculated into a fermentation medium and cultured at 37 °C for 72 h for subsequent screening and analysis.

### 2.8. High-Throughput Fluorescence-Activated Cell Sorting for Mutants

**Figure 1:**
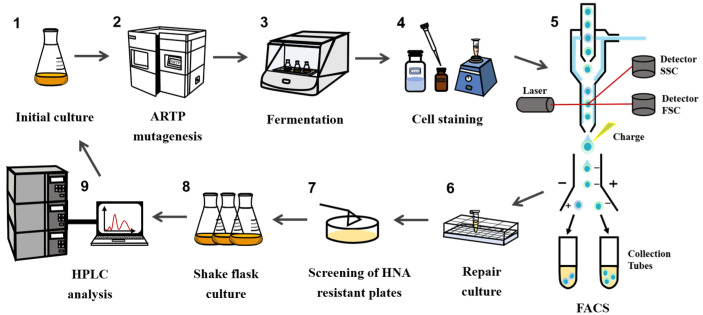
High-throughput screening workflow for high-yielding MK-7 *B. subtilis* mutants.

To ensure the availability of high-yielding MK-7 mutants, a corresponding HTS approach was developed as illustrated in Figure 1, FACS was used to rapidly screen a vast array of mutant libraries generated through ARTP mutagenesis, with forward scatter (FSC) representing cell diameter and side scatter (SSC) representing granularity. A total of four gates were established: P1, P2, P3, and P4. P1 represented the scatter plot of flow-through cells, while aggregated cells were further analyzed in the P2 and P3 gates to eliminate adherent cells. The FI measured in the FITC channel was then used as an indicator to identify strong positive cells within the top 0.1‰ of FI in the P4 gate. These selected mutants were sorted out in ‘Purity’ mode using a sterilized flow cytometer. The target cells were then collected in sterile Eppendorf tubes containing LB medium and immediately placed on ice for preservation. The starting strain used for mutagenesis served as a negative control in each instance.

The cells collected by FACS were revived and cultured at 37 °C and 200 rpm for 1 h to ensure their viability. To increase the likelihood of identifying positive mutant strains, the cells underwent a secondary screening. They were spread on a resistant plate containing 1-hydroxy-2-naphthoic acid (HNA) (90 mg/L) and cultured overnight at 37 °C [36]. Single colonies that were larger and exhibited better growth were selected for further inoculation and fermentation and the MK-7 production levels of these mutant strains were subsequently quantified by HPLC.

### 2.9. Genome Resequencing and Alignment

A combination of second-generation (Illumina TruSeq) and third-generation sequencing technologies (Oxford Nanopore Technologies, ONT, Oxford, UK) was used for whole genome sequencing of the original strain and the high-yielding mutants. The Illumina sequencing samples were prepared following the Standard Illumina TruSeq DNA Sample Preparation Guide and sequenced on the Illumina Nova Seq platform (Illumina, San Diego, CA, USA). The third-generation sequencing was conducted according to the standard protocol provided by ONT and sequenced on the Oxford Nanopore platform. To identify mutant genes in the high-yielding mutants, the whole genome sequencing data of the original strain, SJTU2, was used as the reference genome (GenBank accession number: CP140123), and the sequencing data from the mutants were statistically analyzed to highlight differences. The BWA (version 0.7.12-r1039) mem program was used to align the high-quality filtered data against the reference genome. Duplicate reads were removed using Picard software (version 1.107). Single Nucleotide Polymorphisms (SNPs) and Insertion-Deletion (Indels) were identified by GATK software (version 4.1.9.0) and annotated with ANNOVAR software (latest version) (http://annovar.openbioinformatics.org/, accessed on 27 January 2025). 

### 2.10. Statistical Analysis

Data and figures were mainly processed using GraphPad Prism 8. A one-way analysis of variance (ANOVA) was performed to analyze the data, and Tukey’s test (GraphPad Prism 10 Software, San Diego, CA, USA) was applied for multiple comparisons when evaluating the yield of MK-7 across different mutagenesis cycles.

## 3. Results

### 3.1. Selection of Fluorescent Dyes

The effect of three different fluorescent dyes on FI and growth of *B. subtilis* is shown in Supplementary Figure S1. Compared to the control group, staining with DiBAC4 (3) resulted in only minimal change in FI. In contrast, Rh123 exhibited significant staining effectiveness, with a notable increase in FI, allowing clear differentiation of cells with high fluorescence from the control group. Moreover, staining with DiOC6 (3) produced a substantial enhancement in FI, surpassing that of the two dyes. However, even at minimal concentrations, DiOC6 (3) staining notably prolonged the logarithmic phase of cell growth and resulted in a substantially lower cell concentration in the stationary phase compared to the control group. This result aligns with previous observations [37], indicating that DiOC6 (3) was unsuitable for the subsequent fermentation requirements of this experiment. Consequently, Rh123, possessing excellent fluorescent properties, affordability, and non-toxicity, was chosen as the fluorescent dye for this experiment.

### 3.2. Effect of Pretreatment Methods on Fluorescence Intensity

**Figure 2:**
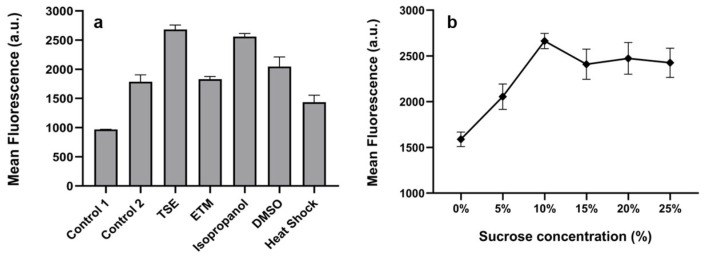
Effect of pretreatment methods on FI. (**a**) Effect of different pretreatment methods on cell staining. Control 1 is cells not stained by Rh123, control 2 is cells stained by Rh123 without pretreatment. (**b**) Effect of different sucrose concentrations of TSE buffer on FI.

To enhance cell membrane permeability and improve the binding of Rh123—a membrane-permeable fluorescent dye that exhibits limited binding to cells—we investigated five different pretreatment methods for *B. subtilis* cells based on previous research, ensuring accurate and reliable results. As illustrated in Figure 2a, the FI significantly increased following treatment with TSE buffer, isopropanol and DMSO. Notably, TSE buffer pretreatment resulted in the highest FI, approximately 1.5 times greater than that of the control group stained without pretreatment.

To achieve better staining results, we investigated the influence of the sucrose concentration of TSE buffer on FI was investigated. As shown in Figure 2b, it is evident that the sucrose concentration of 10% (*w*/*v*) produced the most significant enhancement in the binding efficacy of fluorescent dyes to the cell membrane of *B. subtilis*, making it the chosen pretreatment solution for subsequent experiments. Sucrose treatment has been found to enhance the transport of fluorescent dyes across the cell membrane of *Escherichia coli*, a Gram-negative bacterium [33]. The results in this study indicate that the addition of sucrose significantly affects the Gram-positive bacterium *B. subtilis* cells when treated with TSE buffer. Sucrose may enhance cell staining efficiency by increasing membrane permeability and facilitating the penetration of fluorescent dyes.

### 3.3. Effect of Staining Conditions on Fluorescence Intensity

**Figure 3:**
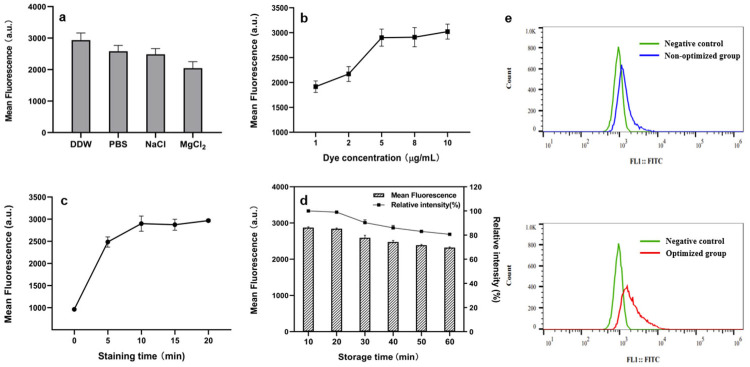
Effect of staining conditions on FI. We investigated the effects of (**a**) different buffers, (**b**) dye concentration, (**c**) staining time, and (**d**) storage time on the FI of cell staining. (**e**) Comparison of staining efficiency before and after optimization. The negative control consisted of cells that were not stained with Rh123. The non-optimized group included cells stained with Rh123 without any pretreatment, while the optimized group comprised cells stained with Rh123 following pretreatment and the optimized protocol.

The fluorescence staining procedure was optimized to achieve increased and more consistent FI. To enhance the binding of fluorescent molecules to the target, we first investigated the effects of commonly used staining buffers—DDW, PBS, 0.9% NaCl, and 1 mM MgCl_2_—on *B. subtilis* cells. As shown in Figure 3a, cells suspended in DDW demonstrated the most effective staining.

Secondly, the optimal dye concentration is crucial for accurate detection results. A concentration that is too low may lead to inadequate staining of the cells, while a concentration that is too high can cause fluorescence self-quenching and complicate bacterial purification. As shown in Figure 3b, a dye concentration of 5 μg/mL resulted in saturation of Rh123 binding to the cells, maintaining a stable fluorescence intensity (FI). Therefore, 5 μg/mL was determined to be the appropriate dye concentration.

Figure 3c illustrates the FI at different staining times. It is noteworthy that a significant increase in fluorescence value was observed after staining for 5 min, demonstrating the ability of the method to rapidly alter cell fluorescence characteristics. However, after 10 min, the growth trend plateaued and the FI stabilized, leading to the selection of a 10-min staining duration.

Prolonged storage causes excited singlet fluorescent molecules to transition to the triplet state, resulting in fluorescence quenching and affecting detection results. In particular, fluorescence loss in cells serves as a critical measure to assess the feasibility of fluorescence methods in long-term screening. Figure 3d illustrates how different storage times affect FI. The results show that after 60 min of storage, the FI remains relatively high at 81% of the initial level, suggesting that this method is suitable for the FACS-based screening processes.

Finally, we successfully optimized the fluorescent staining method for *B. subtilis* cells producing MK-7 and established the following optimal protocol: TSE buffer with 10% sucrose was chosen for pretreatment, DDW was used for resuspension, and Rh123 was used as the fluorescent dye. The dye concentration and staining time were set at 5 μg/mL and 10 min, respectively. The results demonstrate that the optimized protocol significantly enhances staining effectiveness and markedly reduces the false positives caused by bacterial autofluorescence during screening (Figure 3e).

### 3.4. Correlation of Fluorescence Intensity with MK-7 Content

**Figure 4:**
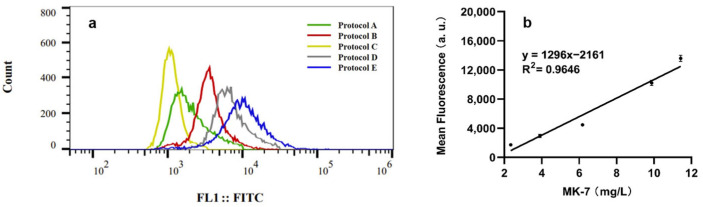
Correlation between MK-7 content and FI. (**a**) FI of *B. subtilis* SJTU2 with five different culture protocols as detailed in Table 1. (**b**) Correlation between MK-7 content and FI of Rh123-stained *B. subtilis* cells.

To investigate the correlation between MK-7 content and FI, five distinct culture protocols were developed, as detailed in Table 1. These protocols varied in carbon sources, nitrogen sources, and culture methods to optimize the MK-7 yield from *B. subtilis* SJTU2. As shown in Figure 4a, the FI of *B. subtilis* varied significantly under different culture conditions, with the intensity ranking from strongest to weakest as follows: E > D > B > A > C. The correlation between MK-7 levels and FI was then analyzed by measuring the MK-7 yield for each protocol.

The yields of MK-7 for protocols A, B, C, D, and E were 3.91 ± 0.09 mg/L, 6.72 ± 0.77 mg/L, 2.34 ± 0.01 mg/L, 9.88 ± 0.13 mg/L, and 11.43 ± 0.07 mg/L, respectively. The results in Figure 4b show a linear correlation between the mean MK-7 yields and the average FI values (R^2^ = 0.9646). This indicates that the FI is proportional to the MK-7 content, confirming the suitability of the method for high-throughput screening of numerous *B. subtilis* mutants that produce MK-7.

### 3.5. ARTP Mutagenesis and High-Throughput Screening of High-Yielding MK-7 Mutants

Given the lack of discernible phenotypic diversity among high-yielding MK-7 strains, establishing a rapid screening technology is challenging. The FI of Rh123 can effectively indicate the level of MK-7 in bacteria. Therefore, FI is used as a sorting criterion to perform high-throughput rapid screening of large mutant libraries. HPLC can then be used for accurate detection of MK-7 levels.

In response to this need, we developed a FACS-based HTS method, as depicted in Figure 1. Individual cells were sorted into the collection tubes based on their FI, with approximately 102–103 cells exhibiting the top 0.1‰ FI being collected. High voltage and flow rate during screening may potentially harm cells. To preserve cell viability, sorted cells were incubated on a shaking table at 37 °C for 1 h to facilitate repair and culture.

**Figure 5:**
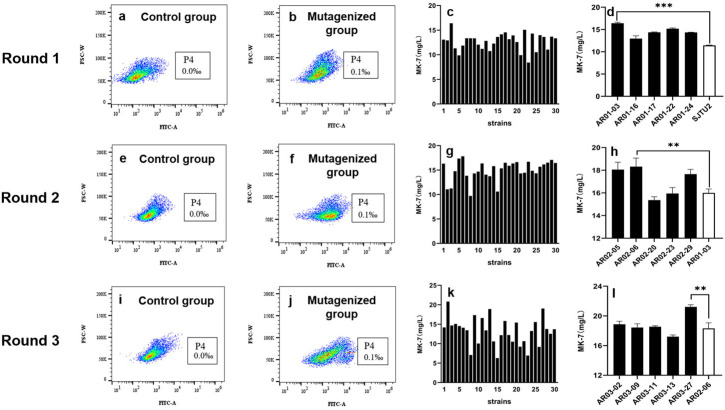
Results of three rounds of FACS and screening of *B. subtilis* mutants producing MK-7. (**a**,**b**,**e**,**f**,**i**,**j**) Dot plots of FACS cell sorting for the control group and mutagenized groups. (**c**,**g**,**k**) Results of 30 mutants selected following repair culture and HNA plate screening. (**d**,**h**,**l**) Retest results for high-yielding mutants. FSC-W: Forward scatter width; FITC-A: The integrated fluorescence signal area. ** *p* < 0.01, *** *p* < 0.001.

In each mutagenesis cycle, the mutant that produced the highest yield of MK-7 was designated as the starting strain (control group) for the next round of mutagenesis. Three cycles of mutagenesis and high-throughput sorting were performed, as shown in Figure 5. Observation of the rounds in Figure 5a,b reveals a notable rightward shift in the cell population following ARTP mutagenesis, indicating an increased presence of positively fluorescing cells within the mutagenized group. This suggests a potential abundance of positive mutant strains.

Figure 5c shows the MK-7 yield of 30 mutants selected after each round of repair culture and HNA plate pre-screening. In the first round, 22 strains yielded more than the starting strain SJTU2 (11.43 ± 0.07 mg/L). Of these, the five mutant strains with the highest yields were further evaluated. As shown in Figure 5d, the strain AR01-03 with the highest yield was successfully identified, with an MK-7 yield of 16.40 ± 0.25 mg/L, representing a 43.48% increase over the starting strain SJTU2.

AR01-03, derived from the first round of mutagenesis, served as the starting strain for the second round of mutagenesis. Subsequently, AR02-06 was selected as the best-performing strain, with an MK-7 yield of 18.32 ± 0.75 mg/L, representing a 60.28% improvement over SJTU2. Finally, AR03-27 (CGMCC 31163) was selected as the best performing with an MK-7 yield of 21.22 ± 0.28 mg/L, representing an 85.65% improvement over SJTU2.

### 3.6. Resequencing Analysis of High-Yielding Mutants

To explore potential factors contributing to the increased MK-7 production in the mutant strains, we performed SNP and InDels analysis. Although no InDels were detected, we observed several large fragment copy number variations in the three high-yielding mutants, so only SNPs are discussed here.

**Figure 6:**
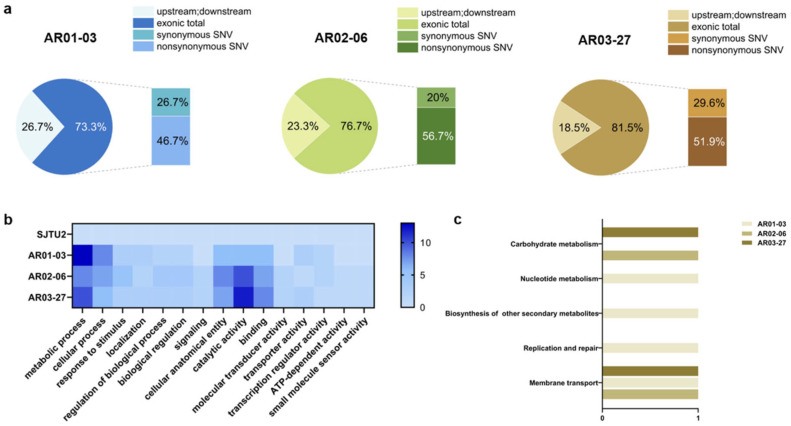
Statistics and annotation of SNPs of three dominant mutants. (**a**) Distribution statistics of SNPs. (**b**) GO Function Classification of SNP mutated genes. (**c**) KEGG Function Classification of SNP mutated genes.

SNP are primarily DNA sequence polymorphisms resulting from single nucleotide variations at the genomic level, including single base conversions, transmutations and similar alterations [38]. As shown in Figure 6a, the statistical distribution of SNPs reveals that over 70% of the mutation sites are located in the coding regions. In particular, the high-yielding mutants AR01-03, AR02-06 and AR03-27 showed 22, 23 and 22 SNP mutations within the coding regions, respectively. Detailed information on SNP mutations in the exon region of the mutants is shown in Supplementary Tables S1–S3.

Annotation and functional cluster analysis were then performed for each SNP mutant gene. The GO functional cluster analysis (shown in Figure 6b) revealed that the SNPs in AR01-03 were predominantly clustered in the “metabolic processes” category. These mutations mainly included genes associated with cell repair or genetic metabolism, such as endonuclease/exonuclease and deoxyribonuclease. Conversely, the mutations observed in AR02-06 and AR03-27 were mainly concentrated in the “catalytic activity” category, predominantly affecting molecular functions related to signal transduction or transporter proteins. Noteworthy examples include the two-component system sensor histidine kinase, the major facilitator superfamily (MFS) transporter, the ATP-binding cassette transporter (ABC transporter), penetrase, and so on. Furthermore, while only a few genes match data in the KEGG database (as depicted in Figure 6c), it is notable that all three high-yielding mutants had mutated genes associated with membrane transport. AR01-03, AR02-06, and AR03-27 have 4, 2, and 2 genes annotated, respectively. For example, GC000777 in AR02-06 and AR03-27 encodes an extracellular solute-binding protein. In AR01-03, GC001710, GC003062, and GC004100 encode aspartyl-phosphate phosphatase YisI, an MFS transporter, and an ABC transporter permease subunit, respectively.

Unfortunately, upon comparison and annotation, we did not identify any rate-limiting enzyme genes associated with the MK biosynthetic pathway (details of SNPs and protein information for the mutants are provided in Tables S1–S3). Nevertheless, based on the GO and KEGG annotation results, a commonality emerged among the three high-yielding mutants: each harbored several mutated genes related to signal transduction or membrane transport. MK is a lipid-soluble compound commonly found in bacterial cell membranes and functions as a membrane protein binding complex within several bacterial electron transport chains [39]. Consequently, MK-7 synthesis is influenced by the state of the cell membrane, and mutations in such genes can facilitate substrate uptake and increase MK-7 production. As a result, membrane engineering has emerged in recent years as a novel approach to promote MK biosynthesis.

A comparison of gene expression changes during MK-7 synthesis in static culture and shaking cultures revealed that the membrane components (GO:0006810, GO:0017000, GO:0008125, GO:0031224, GO:0051234) showed the most significant changes by GO enrichment analysis, highlighting the influence of the cell membrane on MK-7 synthesis [40]. Mutant genes with similar functions were identified in the three high-yielding mutants examined in this study. Table 2 shows the SNP-mutated genes associated with membrane components in these high-yielding mutants, which play a key role in increasing the MK-7 yield of the mutant. Further analysis of the gene mutations related to the electron transport chain in the mutant strains showed that in addition to the gene related to membrane component transport, there was a common mutant gene GC000868 in AR02-06 and AR03-27, which could be expressed as an acetoin dehydrogenase complex. The acetoin dehydrogenase complex can catalyze the formation of acetyl-CoA [41], which is known to play an important role in the electron transport chain, and may promote the MK-7 synthesis by influencing electron transfer. Currently, strain genetic engineering primarily targets rate-limiting enzyme genes associated with the MK biosynthetic pathway. However, the identification of multiple SNP-mutated genes related to membrane components in this investigation suggests the potential for new modification methods. These findings can be further validated using genetic engineering techniques in future research efforts.

## 4. Discussion

In this study, we capitalized on the significant effect of MK-7 content on the transmembrane potential to develop a FACS-based HTS method for rapid screening of a large number of *B. subtilis* mutant libraries.

Vitamin K_2_ plays a significant role in the apoptosis of cells by inhibiting mitochondrial depolarization and reactive oxygen species (ROS) accumulation to restore mitochondrial membrane potential and maintain its structure and functional stability [42]. Fluorescence methods are widely employed for assessing relative changes in membrane potential by measuring FI [43]. Rh123 serves as a prominent fluorescent indicator of mitochondrial membrane potential, capable of accumulating in the matrix as lipophilic cations [26]. Additionally, fluorescent dyes DiBAC4 (3) [44] and DiOC6 (3) [45] are commonly utilized for detecting alterations in mean membrane potential. Consequently, these three fluorescent dyes were chosen to compare their staining efficacy in *B. subtilis*. DiOC6 (3) exhibited the highest FI; however, it was found to be extremely toxic to the cells. In contrast, Rh123 demonstrated a significant increase in FI with negligible effects on cell growth. Consequently, Rh123 was selected as the preferred stain.

Specific carriers of Rh123 and DiBAC4 (3), such as isopropanol [46], sucrose [47], glycerol [48], DMSO [49], MgCl_2_ [33] and ethanol [50], have been reported that increase cell membrane permeability and enhance the binding of fluorescent dyes to various strain cell membranes. During the pretreatment and optimization of staining conditions, we discovered that 10% sucrose solution significantly enhanced staining, while MgCl_2_ had minimal impact and even caused a notable decrease in FI. This discrepancy may be attributed to differences in the composition of the bacterial cell wall and the characteristics of membrane ion channels.

MK-7 content has a pronounced impact on the transmembrane potential, and the FI of Rh123 can reflect changes in membrane potential. However, variations in MK-7 are not the only drivers of membrane potential fluctuations, it is important to acknowledge the correlation between MK-7 content and membrane potential changes. The correlation between the mean intracellular MK-7 content and the average FI supports the feasibility of developing a high-throughput FACS-based screening method for *B. subtilis*, aligning with previous observations [21]. In addition to obtaining high-yielding strains, nutrients, and culture conditions also play an important role in improving the yield [15,35,51]. The higher MK-7 production of protocols D and E may be because the static culture can promote the formation of biofilm, which facilitates the synthesis of valuable natural product menaquinone-7(MK-7) of *B. subtilis* [40]. If genetically engineered bacteria are utilized, MK-7 production could be significantly enhanced. However, further testing is needed to determine whether a linear relationship between MK-7 production and fluorescence intensity still exists across a broader range of MK-7 content [15].

In the FACS instrument, multiple parameters of individual cell, including size, granularity, and fluorescence signal, can be analyzed simultaneously, enabling the screening of up to 10^7^ cells per hour. This capability not only enhances screening efficiency but also significantly reduces the effort required to isolate positive strains. Following three rounds of ARTP mutagenesis and HTS, a high-yielding mutant was successfully isolated, exhibiting an 85.65% increase in yield. ARTP mutagenesis presents a powerful and environmentally friendly alternative to traditional mutagenesis methods, characterized by high efficiency and broad applicability [52].

Traditional plate methods are often inefficient and labor-intensive. In contrast, the approach developed in this study enables the preliminary elimination of wild-type and negative mutants through FACS, followed by a streamlined process for the efficient isolation of high-yield mutants.

The HNA-resistant mutant can alleviate the inhibition of 3-deoxy-D-arabino-heptulosonate-7-phosphate (DAHP) synthetase, the first regulatory enzyme in the shikimate pathway, caused by 1, 4-dihydroxy-2-naphthoate (DHNA) and thus increasing the concentration of DHNA, a key intermediate metabolite in the MK-7 metabolic pathway. The MK-7 yield of HNA resistant mutant separately selected by Tsukamoto et al. increased by 156% [35,36].

Traditional mutagenesis techniques do not involve the introduction of foreign DNA, and may not be classified as GMOs in certain jurisdictions. However, the low expression levels of the related enzymes and the complex metabolic network for MK-7 biosynthesis might be important reasons for the low rates of menaquinone biosynthesis [15]. Traditional strain mutagenesis can improve the production ability but with low efficiency [53]. To overcome this, employing varied mutagenesis methods, such as DNA shuffling or error-prone PCR, more diverse mutant libraries can be generated [15,30]. These libraries can also be efficiently screened for target strains using the HTS strategy developed in this study. Furthermore, this approach is versatile and applicable to a wide range of microbial species, enhancing its overall utility. Additionally, the processes within this strategy can be adaptively refined to further enhance screening efficiency.

Point mutations are well-recognized as the cornerstone of evolution [54]. Small-scale nucleotide variations can significantly impact phenotypic changes [55]. In our analysis of SNP mutant genes, no gene encoding rate-limiting enzyme in the MK biosynthetic pathway was identified. However, unexpectedly, three high-yielding mutants exhibited several mutations in genes related to signal transduction or membrane transport. Given that MK-7 synthesis is influenced by the cell membrane and electron transfer processes [40], these SNPs are crucial for enhancing MK-7 production in mutant strains. Currently, genetic engineering of strains primarily targets genes encoding rate-limiting enzymes of the MK biosynthesis pathway. The discovery of multiple SNPs associated with membrane components found in this study suggests potential new methods for modifying strains, representing a potential new direction to enhance MK biosynthesis [56]. Moreover, the mechanism related to the improved performance of MK biosynthesis can be investigated by other omics analysis [57], and then the MK-7 production may be further optimized by reverse metabolic engineering and synthetic biology technology.

## 5. Conclusions

In conclusion, the significant impact of MK-7 levels on the transmembrane potential was utilized to assess the MK-7 content in *B. subtilis* through Rh123 FI. An HTS method was established to identify high-yield MK-7 *B. subtilis*, utilizing FACS for efficient evaluation of mutant libraries. This approach was applied for the first time to libraries generated via ARTP mutagenesis. After three rounds of ARTP mutation followed by HTS, the standout mutant strain AR03-27 was identified, showing an 85.65% increase in MK-7 yield compared to the baseline SJTU2 strain. The screening method demonstrated a 73% success rate in isolating positive mutants, offering a promising strategy for enhancing vitamin K_2_ production at the industrial level. Resequencing analysis revealed that all top-performing mutants carried SNP mutations in genes related to signal transduction and membrane transport, key factors in enhancing MK-7 production. This finding paves the way for further genetic engineering of strains, including the artificial evolution of key enzymes in metabolic pathways and MK-7-associated membrane transport proteins. MK-7 yields can be enhanced by reverse metabolic engineering and synthetic biology techniques.

## Figures and Tables

**Table 1:** The five different culture protocols.

Protocol	Fermentation Medium	Culture Conditions	References
A	Glucose 5 g/L, soluble starch 5 g/L, soy protein 5 g/L, tryptone 10 g/L, MgSO_4_ 5 g/L, K_2_HPO_4_ 1 g/L, NaCl 5 g/L	Cultured at 37 °C in a rotary shaker at 200 rpm for 72 h	[28]
B	Glycerin 53.6 g/L, soy peptone 100 g/L, K_2_HPO_4_ 10 g/L	Cultured at 37 °C in a rotary shaker at 200 rpm for 72 h	[29]
C	Sucrose 10 g/L, soy protein 15 g/L, NaCl 2.5 g/L, MgSO_4_ 4 g/L, K_2_HPO_4_ 0.05 g/L, CaCl_2_ 0.2 g/L, MnSO_4_ 0.6 g/L	Cultured at 37 °C in a rotary shaker at 200 rpm for 72 h	[30]
D	Yeast powder 25.12 g/L, glycerin 66.01 g/L, K_2_HPO_4_ 0.69 g/L	Cultured at 37 °C in a rotary shaker at 200 rpm for 1 h, then 0 rpm for 71 h.	[31,32]
E	Yeast powder 25.12 g/L, glycerin 66.01 g/L, K_2_HPO_4_ 0.69 g/L	Cultured at 37 °C in a rotary shaker at 200 rpm for 3 h, then 0 rpm for 69 h.	[31,32]

**Table 2:** SNP mutated genes associated with membrane component of dominant mutants.

GO ID	GO Function	AR01-03	AR02-06
GO:0006810	Ion transport	GC001710 GC003062 GC004100	GC000777 GC004055
GO:0017000	Intracellular vesicle transport	/	/
GO:0008125	Pancreatic elastase activity	/	/
GO:0031224	Internal cellular membrane	/	GC000209 GC000454 GC004055
GO:0051234	Intracellular transport	GC001710 GC003062 GC004100	GC000777 GC004055

## Data Availability

The original contributions presented in this study are included in the article/Supplementary Material. Further inquiries can be directed to the corresponding author.

## Supplementary Materials

The following supporting information can be downloaded at: https://www.mdpi.com/article/10.3390/microorganisms13030536/s1, Figure S1: Effects of three different fluorescent dyes on *Bacillus subtilis* cells; Table S1: SNPs and protein information in exon region of AR01-03; Table S2: SNPs and protein information in exon region of AR02-06; Table S3: SNPs and protein information in exon region of AR03-27.

## Abbreviations

The following abbreviations are used in this manuscript:

ARTP	Atmospheric room temperature plasma
DDW	Distilled deionized water
DiBAC4 (3)	Bis-(1, 3-dibutylbarbituric acid) trimethylene oxonol
DiOC6 (3)	3, 3′-dihexyloxacarbocyanine iodide
FACS	Fluorescence-activated cell sorting
FI	Fluorescence intensity
HNA	1-hydroxy-2-naphthoic acid
HTS	High-throughput screening
MK	Menaquinone
Rh123	Rhodamine 123
SNP	Single nucleotide polymorphism
