# Supplementary material for: A High-Throughput Screening Strategy for Bacillus subtilis Producing Menaquinone-7 Based on Fluorescence-Activated Cell Sorting
Source: Microorganisms. 2025 Feb 27;13(3):536. doi: 10.3390/microorganisms13030536 (PMC11946230; doi:10.3390/microorganisms13030536)
Supplement: Supplementary file 1 [file microorganisms-13-00536-s001.zip › microorganisms-3476539-supplementary.pdf]

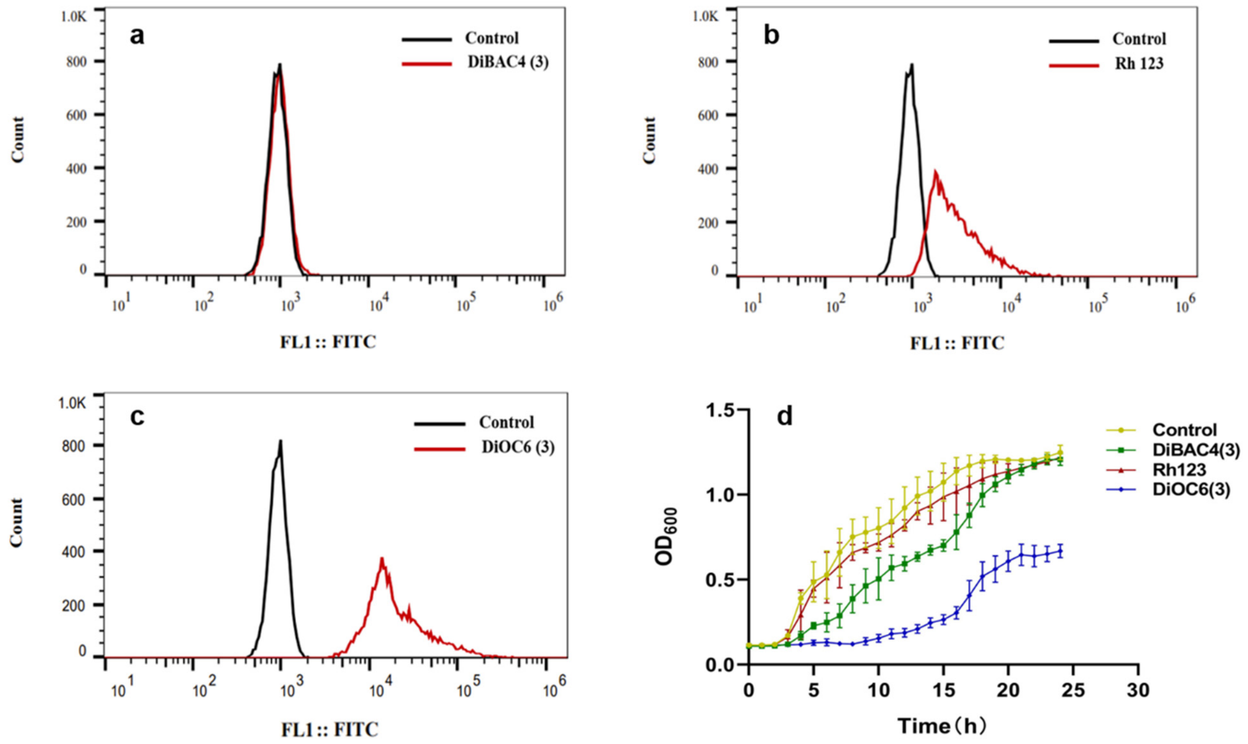

Figure S1. Effects of three different fluorescent dyes on *Bacillus subtilis* cells. (a), (b), (c) Fluorescence intensity after staining with DiBAC4(3), Rh123, and DiOC6(3), respectively. (d) Effects of three different fluorescent dyes on cell growth.

Table S1. SNPs and protein information in coding region of AR01-03.

	Gene	Mutation type	Region	Position	Ref	Alt	Protein
1	GC000540	nonsynonymous SNV	chromosome	535993	A	C	Bifunctional lytictransglycosylase/C40 family peptidase
2	GC000879	synonymous SNV	chromosome	873065	T	G	peptidase cleavage domain-containing protein
3	GC000997	nonsynonymous SNV	chromosome	982955	A	C	Bifunctional lytictransglycosylase/C40 family peptidase
4	GC001142	nonsynonymous SNV	chromosome	1124522	A	C	MarR family transcriptional regulator
5	GC001157	nonsynonymous SNV	chromosome	1140217	T	G	Helicase-exonuclease AddAB subunit AddB
6	GC001599	nonsynonymous SNV	chromosome	1530420	T	G	ATP-dependent deoxyribonuclease subunit A
7	GC001710	nonsynonymous SNV	chromosome	1640587	T	G	Aspartyl-phosphate phosphatase YisI
8	GC002272	nonsynonymous SNV	chromosome	2131444	T	G	Endonuclease III
9	GC002432	nonsynonymous SNV	chromosome	2263230	T	G	Pyrroline-5-carboxylate reductase
10	GC003025	nonsynonymous SNV	chromosome	2818859	T	G	Cysteine synthase
11	GC003062	nonsynonymous SNV	chromosome	2858349	A	C	MFS transporter
12	GC003313	nonsynonymous SNV	chromosome	3110350	T	G	(S)-ureidoglycine--glyoxylate transaminase
13	GC003775	synonymous SNV	chromosome	3519544	A	C	N-acetylmuramoyl-L-alanine amidase
14	GC003876	nonsynonymous SNV	chromosome	3620297	T	G	Ammonium transporter AmtB
15	GC004100	nonsynonymous SNV	chromosome	3835108	T	G	ABC transporter permease subunit
16	GC004173	nonsynonymous SNV	chromosome	3905307	T	G	tRNA nuclease WapA

Table S2 SNPs and protein information in coding region of AR02-06.

	Gene	Mutation type	Region	Position	Ref	Alt	Protein
1	GC000209	nonsynonymous SNV	chromosome	211018	G	C	Two-component system sensor histidine kinase YbdK
2	GC000282	nonsynonymous SNV	chromosome	281599	C	T	Pyroglutamyl-peptidase I
3	GC000454	nonsynonymous SNV	chromosome	462295	G	A	Aldo/keto reductase family oxidoreductase
4	GC000777	nonsynonymous SNV	chromosome	766898	A	G	Extracellular solute-binding protein
5	GC000814	nonsynonymous SNV	chromosome	807757	G	A	Pectate lyase
6	GC000868	nonsynonymous SNV	chromosome	860916	G	A	Acetoin dehydrogenase complex dihydrolipoyl dehydrogenase
7	GC000932	nonsynonymous SNV	chromosome	924488	G	C	Peroxide-responsive transcriptional repressor PerR
8	GC001202	nonsynonymous SNV	chromosome	1182811	G	A	FAD-binding oxidoreductase
9	GC001476	nonsynonymous SNV	chromosome	1413768	C	T	DUF6044 family protein
10	GC001646	nonsynonymous SNV	chromosome	1574551	G	A	CBS domain-containing protein
11	GC001746	nonsynonymous SNV	chromosome	1677468	G	A	Chromosome segregation protein SMC
12	GC002361	nonsynonymous SNV	chromosome	2210915	C	T	Type I signal peptidase
13	GC003394	nonsynonymous SNV	chromosome	3183310	G	C	Spore germination receptor protein GerAC
14	GC003632	nonsynonymous SNV	chromosome	3392465	C	T	Hypothetical protein
15	GC003775	synonymous SNV	chromosome	3519544	A	C	N-acetylmuramoyl-L-alanine amidase
16	GC004005	nonsynonymous SNV	chromosome	3741310	T	G	MFS transporter
17	GC004058	nonsynonymous SNV	chromosome	3794641	C	T	DUF485 domain-containing protein

Table S3. SNPs and protein information in coding region of AR03-27.

	Gene	Mutation type	Region	Position	Ref	Alt	Protein
1	GC000209	nonsynonymous SNV	chromosome	211018	G	C	two-component system sensor histidine kinase YbdK
2	GC000282	nonsynonymous SNV	chromosome	281599	C	T	pyroglutamyl-peptidase I
3	GC000454	nonsynonymous SNV	chromosome	462295	G	A	aldo/keto reductase family oxidoreductase
4	GC000777	nonsynonymous SNV	chromosome	766898	A	G	extracellular solute-binding protein
5	GC000814	nonsynonymous SNV	chromosome	807757	G	A	pectate lyase
6	GC000868	nonsynonymous SNV	chromosome	860916	G	C	acetoin dehydrogenase complex dihydrolipoyl dehydrogenase
7	GC001202	synonymous SNV	chromosome	1182810	G	A	FAD-binding oxidoreductase
8	GC001202	nonsynonymous SNV	chromosome	1182811	G	A	DUF6044 family protein
9	GC001476	nonsynonymous SNV	chromosome	1413768	C	T	Phosphor carrier protein HPr
10	GC001532	nonsynonymous SNV	chromosome	1467353	T	C	CBS domain-containing protein
11	GC001646	nonsynonymous SNV	chromosome	1574551	G	A	DUF669 domain-containing protein
12	GC001919	synonymous SNV	chromosome	1830559	C	T	ABC transporter permease
13	GC002072	nonsynonymous SNV	chromosome	1969583	T	G	RNA methyltransferase
14	GC002910	synonymous SNV	chromosome	2700215	C	T	ABC transporter permease
15	GC003036	nonsynonymous SNV	chromosome	2830928	G	A	hypothetical protein
16	GC003632	nonsynonymous SNV	chromosome	3392465	C	T	N-acetylmuramoyl-L-alanine amidase
17	GC003775	synonymous SNV	chromosome	3519544	A	C	two-component system sensor histidine kinase YbdK
